# Serum procalcitonin and C-reactive protein in the evaluation of bacterial infection in generalized pustular psoriasis^☆^^☆☆^

**DOI:** 10.1016/j.abd.2019.09.022

**Published:** 2019-09-30

**Authors:** Siyu Wang, Zhen Xie, Zhu Shen

**Affiliations:** Department of Dermatology, Institute of Dermatology and Venereology, Sichuan Academy of Medical Sciences & Sichuan Provincial People's Hospital, Chengdu, Sichuan, China

**Keywords:** Bacterial infections, Calcitonin, C-reactive protein, Psoriasis

## Abstract

**Background:**

There is an obvious need for more prompt and specific biomarkers of bacterial infections in generalized pustular psoriasis patients.

**Objective:**

The aim of this study was to evaluate the diagnostic properties and define appropriate cut-off values of procalcitonin and C-reactive protein in predicting bacterial infection in generalized pustular psoriasis patients.

**Methods:**

Sixty-four generalized pustular psoriasis patients hospitalized from June 2014 to May 2017 were included in this retrospective study. The values of procalcitonin, C-reactive protein, details of infection, and other clinical parameters were analyzed.

**Results:**

Receiver operating characteristic curve analysis generated similar areas (*p* = 0.051) under the curve for procalcitonin 0.896 (95% CI 0.782–1.000) and C-reactive protein 0.748 (95% CI 0.613–0.883). A cut-off value of 1.50 ng/mL for procalcitonin and 46.75 mg/dL for C-reactive protein gave the best combination of sensitivity (75.0% for procalcitonin, 91.7% for C-reactive protein) and specificity (100% for procalcitonin, 53.8% for C-reactive protein). Procalcitonin was significantly positively correlated with C-reactive protein levels both in the infected (*r* = 0.843, *p* = 0.040) and non-infected group (*r* = 0.799, *p* = 0.000).

**Study limitations:**

The sample size and the retrospective design are limitations.

**Conclusions:**

The serum levels of procalcitonin and C-reactive protein performed equally well to differentiate bacterial infection from non-infection in generalized pustular psoriasis patients. The reference value of procalcitonin and C-reactive protein applied to predicting bacterial infection in most clinical cases may not be suitable for generalized pustular psoriasis patients. C-reactive protein had better diagnostic sensitivity than procalcitonin; however, the specificity of procalcitonin was superior to that of C-reactive protein.

## Introduction

Generalized pustular psoriasis (GPP) is a rare but severe variant of psoriasis. It is characterized by the sudden onset of widespread erythema, and the superficial, sterile pustules that may coalesce to form lakes of pus, accompanied by high fever and hyperleukocytosis, which can result in significant morbidity and even mortality.^1^ GPP has been widely recognized as a prototypic inflammatory autoimmune disease.^2^ Hereditary, environmental, infectious, and immunological factors have been proposed for the etiopathogenesis of the disease. Infection is a common problem and has become one of the leading causes of mortality in patients with GPP.^3^ It may lead to immune disturbances related to the disease itself or the immunosuppressive therapies.^4^ Infections can be diagnosed by clinical features and positive cultures and/or response to antibiotic therapy. However, a major obstacle for recognizing the presence of infection of GPP patients is that the clinical manifestations of infection are quite similar to those of disease flare. In some patients, both situations – infection and disease flare – can co-exist, making the diagnosis and therapeutic approach a real challenge. Concurrent infection often demands advisable antibiotic treatment with the reduction of the immunosuppressant doses, while active GPP requires upgrade of immune suppression. Considering that the therapies for infection and for active GPP are complete opposites, detecting infections as early and promptly as possible in GPP patients is crucial for the decision-making of treatment strategy to prevent serious health outcomes. Therefore, a simple marker that could help to diagnose bacterial infection of GPP patients at an early stage would be very useful.

Biologic markers such as procalcitonin (PCT) and C-reactive protein (CRP) have been used to distinguish the bacterial infections from non-bacterial infections or disease flares in some autoimmune diseases, such as systemic lupus erythematosus, Behcet's disease, and Henoch-Schönlein purpura, while the results were controversial.^5^, ^6^, ^7^, ^8^, ^9^ PCT is a protein with 116 amino acids and a molecular weight of 13 kDa. It is the precursor in the synthesis of calcitonin (CT) – a calcium-regulating peptide that plays a vital role in calcium homeostasis.^10^ Previous research has demonstrated that serum PCT levels are elevated following bacterial infection, and PCT has been proven to be a useful marker for early diagnosis of bacterial infection and for measuring infectious severity.^11^, ^12^, ^13^, ^14^ CRP is an acute-phase protein that becomes elevated during infections such as pneumonia and sepsis, and it is predominantly produced by liver cells.^15^, ^16^ However, from the viewpoint of the authors, limited information is available on the significance of PCT and CRP in the diagnosis of infection in GPP patients. The aim of the present study was to compare the discriminative diagnostic properties of PCT and CRP for bacterial infection in GPP patients and to define appropriate cut-off values for the recognition of bacterial infection.

## Methods

### Subjects

The authors first reviewed the electronic database of 102 patients whose primary diagnosis was GPP in this hospital from June 2014 to May 2017. The diagnosis of GPP was made according to the diagnostic criteria.^17^ Exclusion criteria: patients who were pregnant, or suffered from systemic diseases (blood, liver, or heart), hypersplenism, acute massive hemorrhage or intoxication, physical/chemical injuries, severe trauma, a history of surgery < 1 month, autoimmune diseases, a history of medicine intake < 3 months (glucocorticoid hormone, immunosuppressive agents, or other drugs that may influence the leukocyte count). Patients with ambiguous bacterial infections (clinically suspected, but lacking positive culture results), nonbacterial infections (viral and/or fungal infection), or *Mycobacterium* tuberculosis infections were also excluded from the study. Based on the above exclusion criteria, 64 GPP patients with complete and detailed medical records were included in this retrospective study. The patients’ information and blood specimens were collected routinely within the first 12 h after their hospitalization. The values of PCT, CRP, leukocyte count, neutrophil ratio, details of infection, and other clinical parameters (including gender, age, duration, body temperature, hospital days, *etc.*) of GPP patients were recorded. The study was approved by the Medical Ethics Committee of this hospital (No. 2017005).

### Measurement of blood index

The leukocyte count and neutrophil ratio were measured using an automated hematology analyzer (Sysmex – Kobe, Japan). The serum levels of CRP and PCT were measured using nephelometry immunoassay (Beckman Coulter – United States) and electrochemiluminescence immunoassay (BRAHMS – Germany), respectively.

### Definition of infection

The diagnosis of bacterial infection was based on the positive culture results of pathogenic microorganism. The presence of an infection was confirmed by the presence of a positive pathogen test from various specimens (blood, sputum, pus, stool, urine, or other cultures). Bacterial infection was defined as a positive culture for aerobic or anaerobic microorganisms, with identification of the bacterial species. A positive response to the standard antibacterial therapy was also used to support the diagnosis of bacterial infection.

### Statistical analysis

The statistical analysis was performed using SPSS v. 23.0 (SPSS – United States). A chi-squared test was performed to determine qualitative variables. Quantitative variables with a non-normal distribution were expressed as median (range), and quantitative variables with a normal distribution were expressed as mean ± standard deviation. Analysis of variance was used for comparing differences in data between normal distribution groups, while the Mann–Whitney test was used between non-normal distribution groups. Receiver operating characteristic (ROC) curves were plotted to evaluate the diagnostic power of the serum levels of PCT and CRP by determining the area under the curve (AUC). Sensitivity, specificity, positive predictive value (PPV), and negative predictive value (NPV) of PCT and CRP were also calculated. Correlation coefficients between groups were obtained by calculating the Pearson coefficient correlation (normal distribution) or the Spearman coefficient correlation (non-normal distribution). A value of *p* < 0.05 was considered significant, and all tests were two-tailed.

## Results

### Patient characteristics

Demographic and clinical characteristics of the study patients are presented in Table 1. A total of 64 GPP patients were enrolled, and half of the patients were male (*n* = 32, 50%). Twelve patients (18.75%) were diagnosed as infected by bacteria. The infected group included five cases with urinary tract infection (three cases were *Escherichia coli*, and one case each of *Bacillus proteus* and *Streptococcus uberis*, respectively), three with pneumonia (two cases were *Streptococcus pneumoniae*, one case was *Haemophilus influenzae*), and four with skin and soft tissue bacterial infections (all cases were *Staphylococcus aureus*). There was no significant difference in gender distribution (male/female 7/5 *vs.* 25/27, *p* = 0.522); age (49.83 ± 14.88 *vs.* 39.88 ± 16.22, *p* = 0.057); duration (96 [24–465] *vs.* 72 [1–360], *p* = 0.369); or hospital day (13.5 [6–20] *vs.* 12.5 [6–26], *p* = 0.782) since diagnosis of GPP between infected and non-infected groups of patients. The body temperature (37.4 [36.7–38.9] *vs.* 36.8 [36.0–39.2]; *p* = 0.002) and the serum levels of CRP (68.35 [32.94–162.83] *vs.* 45.65 [0.50–161.95], *p* = 0.008) and PCT (2.25 [0.12–6.26] *vs*. 0.15 [0.05–1.02], *p* = 0.000) in the infected group were greater than those in the non-infected group, and the differences were statistically significant.

**Table 1:** Characteristics, clinical parameters, and blood indices of generalized pustular psoriasis (GPP) patients.

Characteristics of patients	Infected group	Non-infected group	*p*-Value
*Sex*			
Male	7	25	0.522
Female	5	27	
Age, average ± SD (years)	49.83 ± 14.88	39.88 ± 16.22	0.057
Duration (months), median (range)	96 (24–465)	72 (1–360)	0.369

*Bacterial infection*			
Urinary tract infection	5		
Pneumonia	3		
Skin and soft tissue bacterial infections	4		
Body temperature (°C), median (range)	37.4 (36.7–38.9)	36.8 (36.0–39.2)	0.002
Hospital day, median (range)	13.5 (6–20)	12.5 (6–26)	0.782
Leukocyte count, average ± SD (10^9 L^−1^)	13.27 (9.38–23.32)	10.01(3.27–17.50)	0.001
Neutrophil ratio, median (range)	0.79 (0.71–0.89)	0.78 (0.49–0.90)	0.092
PCT (ng/mL), median (range)	2.25 (0.12–6.26)	0.15 (0.05–1.02)	0.000
CRP (mg/L), median (range)	68.35 (32.94–162.83)	45.65 (0.50–161.95)	0.008

CRP, C-reactive protein; PCT, procalcitonin.

### PCT and CRP in the infected and non-infected groups

**Figure 1:**
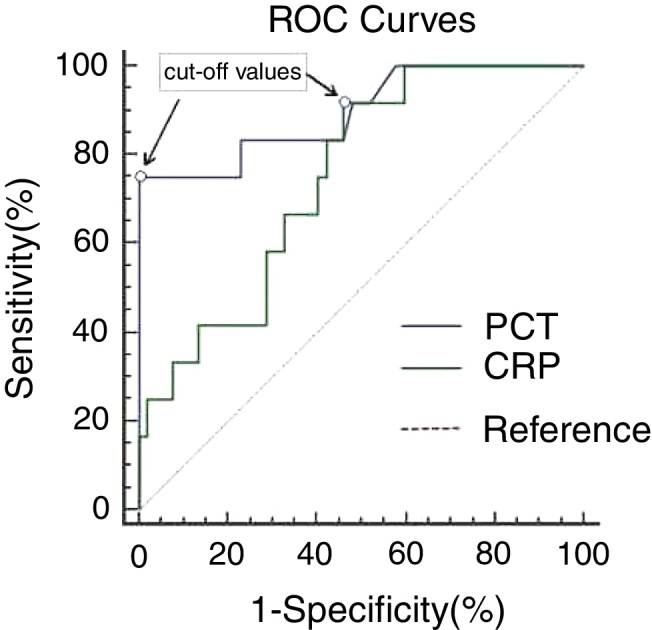
ROC curves of PCT and CRP in GPP patients. ROC curves comparing sensitivity and specificity of procalcitonin (PCT) and C-reactive protein (CRP) levels to diagnose bacterial infection (12 with bacterial infection and 52 without infection). The cut-off values with the best combination of sensitivity and specificity for PCT and CRP were 1.50 ng/mL and 46.75 mg/L, respectively.

Both PCT (2.25 *vs.* 0.15 ng/mL, *p* = 0.000) and CRP (68.35 *vs.* 45.65 mg/dL, *p* = 0.008) were significantly higher in GPP patients with bacterial infection compared to those without infection. ROC curve analysis generated similar areas (*p* = 0.051) under the curve for PCT (0.896, 95% CI: 0.782–1.000) and CRP (0.748, 95% CI: 0.613–0.883), as shown in Fig. 1 and Table 2. A cut-off value of 1.50 ng/mL for PCT gave the best combination of sensitivity (75.0%) and specificity (100%), with PPV and NPV of 100% and 94.5%, respectively. Decreasing the PCT threshold to 0.115 ng/mL resulted in 100% sensitivity, but specificity was greatly reduced to 42.3%. A cut-off level of 46.75 mg/dL for CRP gave a combination of 91.7% sensitivity and 53.8% specificity, with PPV and NPV of 31.4% and 96.6%, respectively. Decreasing the CRP threshold to 31.67 ng/mL resulted in 100% sensitivity, but specificity was greatly reduced, to 40.4%. To achieve 100% specificity, the CRP threshold would need to be increased to 162.23 mg/dL.

**Table 2:** Diagnostic properties and appropriate cut-off values of procalcitonin (PCT) and C-reactive protein (CRP) for diagnosing bacterial infection of generalized pustular psoriasis (GPP) patients.

	PCT	CRP
Cut-off value	1.50 ng/mL	46.75 mg/dL
Sensitivity, %	75	91.7
Specificity, %	100	53.8
Positive predictive value, %	100	31.4
Negative predictive value, %	94.5	96.6
Asymptotic 95% confidence interval	0.782–1.000	0.613–0.883
Area under the receiver operating curve	0.896	0.748

### Factors associated with PCT and CRP variations in subgroups

In the non-infected group, PCT and CRP were both positively correlated with age (*r* = 0.930, *p* = 0.000 for PCT, *r* = 0.691, *p* = 0.000 for CRP), leukocyte count (*r* = 0.835, *p* = 0.000 for PCT, *r* = 0.989, *p* = 0.000 for CRP), and neutrophil ratio (*r* = 0.962, *p* = 0.000 for PCT, *r* = 0.843, *p* = 0.001 for CRP); however, CRP (*r* = 0.500, *p* = 0.000), but not PCT (*r* = −0.044, *p* = 0.730), was positively correlated with body temperature in the non-infectious group. In the infected group, PCT (*r* = 0.962, *p* = 0.000) and CRP (*r* = 0.799, *p* = 0.000) were positively correlated with body temperature; however, neither PCT nor CRP was correlated or above moderately correlated (*r* < 0.5) with age (*r* = 0.281, *p* = 0.025 for PCT, *r* = 0.367, *p* = 0.003 for CRP), leukocyte count (*r* = −0.266, *p* = 0.034 for PCT, *r* = 0.070, *p* = 0.584 for CRP), or neutrophil ratio (*r* = 0.209, *p* = 0.098 for PCT, *r* = −0.059, *p* = 0.646 for CRP) in the infected group. PCT was positively correlated with CRP levels in the infected (*r* = 0.843, *p* = 0.040) and the non-infected group (*r* = 0.799, *p* = 0.000). See Table 3 for more details.

**Table 3:** Correlation between serum levels of procalcitonin (PCT)/C-reactive protein (CRP) and clinical parameters in generalized pustular psoriasis (GPP) patients.

	Infected group				Non-infected group			
	PCT		CRP		PCT		CRP	
	*r*	*p*-Value	*r*	*p*-Value	*r*	*p*-Value	*r*	*p*-Value
Age	0.281	0.025	0.367	0.003	0.930	0.000	0.691	0.000
Body temperature	0.962	0.000	0.799	0.000	−0.044	0.730	0.500	0.000
Leukocyte count	−0.266	0.034	0.070	0.584	0.835	0.000	0.989	0.000
Neutrophil ratio	0.209	0.098	−0.059	0.646	0.962	0.000	0.843	0.001
PCT	–	–	0.843	0.040	–	–	0.799	0.000

## Discussion

It is well known that flaring GPP patients are immunocompromised by the disease itself or by concomitant immunosuppressive therapy, and flares may sometimes mimic infections, with leukocytosis, fever, and chills.^4^ Prompt identification of early-stage bacterial infection is very important, since appropriate etiological treatment and avoidance of unnecessary antimicrobial therapy can not only reduce the morbidity, mortality, and costs to patients, but also can reduce the emergence of antibiotic-resistant bacteria. Microbiologic culture takes at least 24–48 h, and negative cultures do not exclude the presence of infection.^18^ Therefore, there is an obvious need for more specific biomarkers of bacterial infections in GPP patients.

PCT, the precursor of the hormone calcitonin, is produced by C-cells of the thyroid gland or neuroendocrine cells in the lung or intestine, in response to cytokines such as interleukin-6 (IL-6) and tumor necrosis factor-alpha (TNF-α), and its level in the blood of healthy people is low.^19^, ^20^ The level of PCT rises remarkably during bacterial infections, reaches a plateau at between 12 and 48 h, and then drops if the stimulus stops, whereas low levels are detected during viral infections or non-infectious febrile conditions.^21^, ^22^ CRP, synthesized by the liver, is one of the acute-phase inflammation proteins, whose baseline concentration in healthy individuals is below 1 mg/L. The level of serum CRP increases six to eight hours after stimulation by IL-6, with a half-life of 20 to 24 h.^23^

In the present study, it was demonstrated that the PCT and CRP levels of GPP patients in the infected-group were significantly higher than those of non-infected group, suggesting that both PCT and CRP can be used to differentiate potential bacterial infection from the flare of GPP itself, which was further confirmed by calculating the areas under the ROC curves. Based on the areas under the ROC curves, PCT (AUC = 0.896) and CRP (AUC = 0.748) performed equally well (*p* = 0.051) to differentiate bacterial infection from non-bacterial infection in GPP patients in the present study. The cut-off values that showed the best combination of sensitivity and specificity, using the laboratory methods stated, were 1.50 ng/mL for PCT and 46.75 mg/dL for CRP. As for these thresholds, CRP had better sensitivity (91.7%) than PCT (75%), but the diagnostic specificity of PCT (100%) was superior to that of CRP (42.9%). Both tests generated high negative predictive values (94.5% for PCT and 96.6% for CRP). Therefore, these cut-offs values should be used to “exclude” bacterial infection. In consistence with the findings of earlier records,^24^, ^25^ the current study has indicated that the diagnostic specificity of PCT in detecting bacterial infection is superior to CRP in GPP patients. It may be attributed to the fact, proven by previous studies, that PCT did not appear to be pivotally influenced by viral infections, autoimmune or allergic disorders, immunosuppressives, or steroids.^26^, ^27^, ^28^ Nonetheless, CRP is a biomarker of inflammation rather than a biomarker of infection. Its level rises in most pathological cases associated with inflammation, such as bacterial/viral infections, trauma, systemic disease flare and post-surgical period.^29^

To completely “include” patients with bacterial infection, the cut-off values that gave 100% sensitivities were generated by PCT > 0.115 ng/mL and CRP > 31.67 mg/dL, whose values were higher than the PCT (0.05 ng/mL) and CRP (3 mg/dL) guidance values in predicting suspicious infection in clinical practice. Therefore, it is feasible that the reference values of PCT and CRP, those applied to predicting probable infection in most clinical cases, may not be suitable for GPP patients. However, it should also be noted that the accompanying low specificities (42.3% for PCT, 40.4% for CRP) and high rates of false positive may arise if cut-off values of PCT and CRP with 100% sensitivity are used as markers for diagnosing bacterial infection.

It has been a controversial issue whether PCT or CRP was significantly correlated with age in the infected or non-infected population, and the present study implied that PCT/CRP was above moderately (*r* > 0.5) positively correlated with age in the infected group, but not in the non-infected group (*r* < 0.5).^27^, ^30^, ^31^, ^32^ In the present results, it is noteworthy that, in both the infected and non-infected group, a positive correlation was found between levels of CRP and PCT, and the cut-off values of CRP and PCT for differentiating bacterial infection from active disease phase are higher than those of other autoimmune diseases. It may be partly due to the fact that some cytokines, including IL-6, may be induced by Th2 immune response of GPP, which may simultaneously stimulate the synthesis levels of PCT and CRP.^33^ The present research has also shown that PCT and CRP, only in the non-infected group, were positively correlated with leukocyte count and neutrophil ratio, respectively. The previous research has explained that the up-regulation of leukocytes and neutrophils represent the responses of body to systemic inflammation from GPP.^34^ The positive correlations between CRP/PCT and leukocyte count or neutrophil ratio in the non-infected group may reflect such a possibility, so that CRP and PCT (without infection) indirectly influenced by inflammatory mediators (including TNF-α, IL-6, *etc.*) could be considered as the inflammation biomarkers of GPP. If the patient suffered from a concurrent infection, the increased CRP could activate the classical complementary metabolic pathway and bind to bacteria, with subsequent activation of leukocyte-mediated cytotoxicity, thus it is assumed that it might change the correlations between CRP and leukocyte count/neutrophil ratio in the preceding non-infected condition.^35^ Meanwhile, PCT that could be directly influenced by the bacterial endotoxins and lipopolysaccharides was no longer correlated with leukocyte count or neutrophil ratio.^36^ On the other hand, previous studies have found that PCT is produced by different cell types (including thyroid C-cells, neuroendocrine cells, peripheral blood mononuclear cells, and parenchymal tissues, *etc.*) under physiological and pathological conditions.^37^, ^38^, ^39^ However, the site of PCT production in GPP and whether it has any relation to leukocyte remains unknown. Moreover, it is not clear whether the increased PCT is merely an epiphenomenon or rather is related to the pathogenesis of GPP. In addition, CRP (both in the infected and non-infected group) and PCT (only in the infected group) were positively correlated with body temperature; however, the results about correlations between CRP/PCT and body temperature in correlated groups still cannot be explained. Therefore, further studies need to be conducted.

This study has a few limitations. Firstly, as a retrospective design, there might have selection bias. Secondly, patients were classified into the infected groups based on positive pathogen culture, so the possibility of a false negative culture result exists. There were four patients who were clinically suspected to have local infection but whose cultures were negative, and all four of these ambiguous cases were excluded from this study in order to eliminate this limitation. The measurement techniques and diagnostic criteria for infection used in this study were consistent across all participants. Finally, this study's sample population was not very large due to the rarity of GPP as well as the strict exclusion criteria, and the insufficient number of patients in some of the analysis subgroups made it difficult to draw robust conclusions. Nevertheless, to the best of the authors’ knowledge, there are no reports about the diagnostic properties of PCT and CRP for bacterial infection in GPP patients. Further studies concerning a larger number of cases will be necessary.

## Conclusion

To conclude, in the present study, both PCT and CRP have been demonstrated to have the capacity to differentiate potential bacterial infection from the flare of GPP itself. The reference value of PCT and CRP applied to predicting bacterial infection in most clinical cases may not be suitable for GPP patients. A positive correlation was found between levels of CRP and PCT, and cut-off values of CRP and PCT for differentiating bacterial infection from disease flare are higher than those of other autoimmune diseases. PCT and CRP performed equally well to differentiate bacterial infection from non-bacterial infection in GPP patients based on the areas under the ROC curves. CRP had better diagnostic sensitivity than PCT; however, the specificity of PCT was superior to that of CRP.

## Financial support

The study was supported by the Clinical Research and Key Transformation Project of Sichuan Academy (2016LZ02) and the Special Foundation for Young Scientists (2016QN15) of the Sichuan Academy of Medical Sciences & the Sichuan Provincial People's Hospital.

## Author's contributions

Siyu Wang: Statistical analysis; approval of the final version of the manuscript; conception and planning of the study; elaboration and writing of the manuscript; obtaining, analyzing and interpreting the data; effective participation in research orientation; intellectual participation in propaedeutic and/or therapeutic conduct of the cases studied; critical review of the literature; critical review of the manuscript.

Zhen Xie: Statistical analysis; approval of the final version of the manuscript; conception and planning of the study; obtaining, analyzing and interpreting the data; effective participation in research orientation; critical review of the literature; critical review of the manuscript.

Zhu Shen: Statistical analysis; approval of the final version of the manuscript; conception and planning of the study; elaboration and writing of the manuscript; effective participation in research orientation; intellectual participation in propaedeutic and/or therapeutic conduct of the cases studied; critical review of the literature; critical review of the manuscript.

## Conflicts of interest

None declared.
